# Annexin A1 promotes the progression of bladder cancer via regulating EGFR signaling pathway

**DOI:** 10.1186/s12935-021-02427-4

**Published:** 2022-01-06

**Authors:** Piao Li, Lingling Li, Zhou Li, Shennan Wang, Ruichao Li, Weiheng Zhao, Yanqi Feng, Shanshan Huang, Lu Li, Hong Qiu, Shu Xia

**Affiliations:** 1 Department of Oncology, Tongji Hospital, Tongji Medical College of Huazhong University of Science and Technology, 1095 Jie Fang Avenue, Wuhan, Hubei 430030 People’s Republic of China; 2 Department of Geriatric, Tongji Hospital, Tongji Medical College of Huazhong University of Science and Technology, Wuhan, Hubei 430030 People’s Republic of China

**Keywords:** Annexin A1, Bladder cancer, Progression, EGFR signaling

## Abstract

**Background:**

Bladder cancer (BLCA) is one of the most common malignancies worldwide. One of the main reasons for the unsatisfactory management of BLCA is the complex molecular biological mechanism. Annexin A1 (ANXA1), a Ca^2+^-regulated phospholipid-binding protein, has been demonstrated to be implicated in the progression and prognosis of many cancers. However, the expression pattern, biological function and mechanism of ANXA1 in BLCA remain unclear.

**Methods:**

The clinical relevance of ANXA1 in BLCA was investigated by bioinformatics analysis based on TCGA and GEO datasets. Immunohistochemical (IHC) analysis was performed to detect the expression of ANXA1 in BLCA tissues, and the relationships between ANXA1 and clinical parameters were analyzed. In vitro and in vivo experiments were conducted to study the biological functions of ANXA1 in BLCA. Finally, the potential mechanism of ANXA1 in BLCA was explored by bioinformatics analysis and verified by in vitro and in vivo experiments.

**Results:**

Bioinformatics and IHC analyses indicated that a high expression level of ANXA1 was strongly associated with the progression and poor prognosis of patients with BLCA. Functional studies demonstrated that *ANXA1* silencing inhibited the proliferation, migration, invasion and epithelial–mesenchymal transition (EMT) of BLCA cells in vitro, and suppressed the growth of xenografted bladder tumors in vivo. Mechanistically, loss of *ANXA1* decreased the expression and phosphorylation level of EGFR and the activation of downstream signaling pathways. In addition, knockdown of *ANXA1* accelerated ubiquitination and degradation of P-EGFR to downregulate the activation of EGFR signaling.

**Conclusions:**

These findings indicate that ANXA1 is a reliable clinical predictor for the prognosis of BLCA and promotes proliferation and migration by activating EGFR signaling in BLCA. Therefore, ANXA1 may be a promising biomarker for the prognosis of patients with BLCA, thus shedding light on precise and personalized therapy for BLCA in the future.

**Supplementary Information:**

The online version contains supplementary material available at 10.1186/s12935-021-02427-4.

## Introduction

Bladder cancer (BLCA) is one of the most common malignancies worldwide, with approximately 5490,000 new cases and 200,000 deaths per year, and is generally classified into two major groups based upon depth of invasion: non-muscle-invasive bladder cancer (NMIBC) and muscle-invasive bladder cancer (MIBC) [1, 2]. Approximately 80% of bladder cancer patients are diagnosed with NMIBC, and the remaining 20% present with MIBC [2]. Although transurethral resection followed by Bacillus Calmette–Guérin or intravesical chemotherapeutic agents have excellent efficacy for MNIBC, more than 50% of patients experience recurrence [3–5]. Compared with NMIBC, MIBC exhibits higher aggressiveness, metastasis rate, and mortality [2]. The current standard of clinical management for MIBC is radical cystectomy with pelvic lymph node dissection after neoadjuvant platinum-based chemotherapy [6, 7]. Even though neoadjuvant chemotherapy, antibody–drug conjugates, target therapies, and immunotherapy have been introduced and shown to be beneficial for some bladder cancer patients, improving the prognosis of advanced BLCA patients still faces severe challenges [7–9]. Hence, it is imperative to further study the molecular mechanism of the tumorigenesis and progression of bladder cancer and develop novel therapeutic targets for bladder cancer.

Annexin A1 (ANXA1), the first member of the annexin superfamily, is a Ca^2+^-dependent phospholipid-binding protein with two distinct regions: a variable N-terminal domain and a conserved C-terminal domain [10]. ANXA1 was initially identified as a glucocorticoid-regulated anti-inflammatory protein that regulates innate and adaptive immune responses [11–13]. Subsequently, it has been discovered to play various roles in tumorigenesis and progression, including proliferation, migration, invasion, apoptosis, and differentiation [14, 15]. In addition, more comprehensive and in-depth studies have found that the expression pattern and function of ANXA1 in different tumor types are tissue-specific. It has been reported that ANXA1 is downregulated in head and neck squamous cell carcinoma, prostate carcinoma, and esophageal carcinoma, relating to differentiation grade [16–18]. While in lung adenocarcinoma, gastric cancer, hepatocellular carcinoma, melanoma, and pancreatic cancer, ANXA1 is upregulated and associated with poor prognosis [19–23]. To date, the role of ANXA1 in BLCA remains unclear, and its expression pattern is controversial. Lunbiao Cui et al. showed that ANXA1 was downregulated in human bladder transitional cell carcinomas [24]. In contrast, several other studies indicated that ANXA1 was upregulated in BLCA and correlated with high pathological grade and tumor progression [25–27]. The exact expression pattern, biological functions, and molecular mechanism of ANXA1 in BLCA have yet to be elucidated. Hence, further research is needed to gain a better understanding of ANXA1 and to assess its potential as a diagnostic or prognostic biomarker and therapeutic target for bladder cancer.

In this study, we first investigated the relationship between the expression level of ANXA1 and clinicopathological features in BLCA. Next, we systematically examined the biological functions of ANXA1 in BLCA cells and found that loss of *ANXA1* effectively suppressed the growth of BLCA cells in vitro and in vivo. Subsequently, the possible molecular mechanism of ANXA1 in BLCA was explored. In summary, our findings demonstrated that ANXA1 promoted tumor progression by activating EGFR signaling, which revealed that ANXA1 might be a prognostic predictor and underlying therapeutic target for BLCA.

## Materials and methods

### Bioinformatic analysis

The gene expression and clinical information of BLCA samples were acquired from The Cancer Genome Atlas (TCGA) (https://tcga-data.nci.nih.gov/tcga/) and Gene Expression Omnibus (GEO) (https://www.ncbi.nlm.nih.gov/geo/) databases. The TCGA dataset includes 414 BLCA samples, of which 407 samples have survival information. The GEO dataset (GSE13507) includes 165 primary BLCA samples. These datasets were used to analyze the correlation between ANXA1 and clinicopathological features. Samples in the TCGA dataset were divided into the low *ANXA1* group (n = 200) and high *ANXA1* group (n = 207) on the basis of the optimal cutoff value determined by X-tile software [28]. In the same way, samples in the GEO dataset were divided into the low ANXA1 group (n = 147) and high ANXA1 group (n = 18). The data of mRNA and protein expression were downloaded from cBioPortal for Cancer Genomics website (http://www.cbioportal.org/). According to the ANXA1 protein expression level, the samples from cBioPortal with the lowest twenty percent and highest twenty percent ANXA1 were regarded as the low ANXA1 (n = 23) and high ANXA1 (n = 23) groups, respectively. The unpaired t-test was used to determine the differentially expressed proteins in the high ANXA1 and low ANXA1 groups. Pearson correlation analysis was conducted to study the relationship between ANXA1 and EGFR at the gene and protein expression levels. Genes of TCGA bladder cancer samples were pre-ranked and analyzed for Gene Set Enrichment Analysis (GSEA, v4.1.0) to generate enrichment plots for target gene sets. Normalized enrichment scores (NES) of different target gene sets were calculated, and FDR < 0.05 was regarded as statistically significant.

### Bladder cancer specimens and immunohistochemistry (IHC)

A tissue array of BLCA (HCol-Ade180Sur-07, Shanghai, China) with clinical information was purchased from Shanghai Outdo Biotech for IHC analysis. Paraffin-embedded tissue sections were subjected to deparaffinization, antigen retrieval, and blockade of nonspecific binding, followed by incubation in primary antibodies overnight at 4 °C. Specific primary antibodies against Annexin A1 (1:200, 32934, Cell Signaling Technology, Danvers, MA), P-EGFR (1:100, 3777, Cell Signaling Technology, Danvers, MA), and Ki-67 (1:200, 9027, Cell Signaling Technology, Danvers, MA) were used for IHC. After incubation with secondary antibodies for 30 min at room temperature, the slides were stained with 3,3-diaminobenzidine and then counterstained with hematoxylin. The immunohistochemical score (IHC score) based on the staining intensity and percentage of positive cells was calculated to evaluate the protein expression level of ANXA1 [29].

### Cell culture and reagents

The BLCA cell lines (T24 and 5637) were purchased from the Cell Bank of Type Culture Collection of Chinese Academy of Sciences (Shanghai, China), confirmed to be mycoplasma negative, and identified by short tandem repeat (STR) profiling. 5637 cells were cultured in RPMI 1640 medium (KeyGEN BioTECH, Jiangsu, China) supplemented with 10% FBS (Gibco, Carlsbad, USA), and the T24 cell line was cultured in McCoy's 5A medium (KeyGEN BioTECH) supplemented with 10% FBS (Gibco, Carlsbad, USA). Cells were maintained in a humidified 37 °C incubator with 5% CO_2_ and 95% air. After growing to 80% confluence, cells were digested by trypsin (Biosharp, Shenzhen, China) for passage. Recombinant human epidermal growth factor (EGF, HY-P7109), cycloheximide (CHX, HY-12320), and MG132 (HY-13259) were purchased from MedChemExpress (Monmouth Junction, NJ). All reagents were stored and used in accordance with the instructions of the manufacturer.

### Lentivirus transfections

Short hairpin RNA (sh-RNA) against *ANXA1* and negative control nontargeting sequences were constructed in the pCLenti-U6-shRNA-EF1-Luc2-F2APuro-WPRE vector (OBiO Technology). BLCA cell lines (5637 and T24) were transfected with sh-ANXA1 and negative control (NC) lentivirus for 24 h, followed by treatment with 2 μg/mL puromycin to screen for stably transfected cells.

### Western blot analysis

Western blotting was performed as previously described [29]. Protein samples were separated by SDS–PAGE electrophoresis and transferred onto polyvinylidene fluoride (PVDF) membranes. Then, the PVDF membranes with proteins were incubated with 5% nonfat milk, specific primary antibodies at 4 °C overnight, and secondary antibodies (1:5000, BA1050, BA1054, Boster, Wuhan, China) in sequence and detected using ECL reagent. The primary antibodies were as follows: anti-Annexin A1 (1:1000, 32,934, Cell Signaling Technology), anti-E-cadherin (1:1000, ab231303; Abcam, Cambridge, U.K.), anti-vimentin (1:1000, ab20346; Abcam), anti-MMP9 (1:1000, ab76003; Abcam), anti-EGFR (1:1000, 66,455–1-Ig; Proteintech), anti-phospho-EGFR (1:1000, 3777, Cell Signaling Technology), anti-AKT(1:1000, 4685, Cell Signaling Technology), anti-phospho-AKT (1:1000, 4060, Cell Signaling Technology), anti-ERK (1:1000, 4659, Cell Signaling Technology), anti-phospho-ERK (1:1000, 4370, Cell Signaling Technology), anti-STAT3, anti-phospho-STAT3, anti-GAPDH (1:10,000, 60004–1-Ig; Proteintech), and anti-alpha tubulin(1:10,000, 66031-1-Ig; Proteintech) antibodies.

### Quantitative Real-Time PCR (qRT–PCR)

The detailed experimental procedures of qRT–PCR were as described in our previous study [29]. Total RNA was isolated and synthesized cDNA. qRT–PCR analysis was performed to measure the gene expression of *EGFR* and *ANXA1*. The sequences of primers are shown in Additional file 4. Table S1. Relative gene expression levels were normalized against the internal control using the 2^−ΔΔCT^ method.

### CCK-8 and EdU assays

Cells were seeded into 96-well plates (3000 cells per well). The time at which cells adhere to the bottom is recorded as the 0 timepoint. After culturing cells for different durations, CCK-8 (10 μl per well) was added. Cells with CCK-8 were protected from light at 37 °C for 2 h. The absorbance of each well at a wavelength of 450 nm (OD450) was measured by a microplate reader (BioTek, Winooski, VT).

The proliferation ability of cells was detected using an EdU kit (RiboBio, China). Cells were seeded into 96-well plates (5000 cells per well). After attachment, cells were incubated with 50 μM EdU reagent at 37 °C for 3 h, fixed with 4% paraformaldehyde for 15 min and treated with 0.5% Triton X-100 for 20 min. The cells were incubated with 1 × Apollo® reaction cocktail in the dark for 30 min and stained with Hoechst 33,342 for 20 min. EdU-positive cells were observed under a fluorescence microscope.

### Colony formation and cell cycle assays

For the colony formation assay, cells were seeded in 6-well plates (200 cells per well), cultured with complete medium for two weeks, fixed with 4% formaldehyde and stained with 0.1% crystal violet. The clones of each well were counted and photographed.

For the cell cycle assay, cells were collected, fixed with 70% cold methanol overnight, and stained with propidium iodide (PI) solution containing RNase away from light for 30 min at 37 °C. Cell cycle distribution was detected using Attune NxT flow cytometry (Thermo Fisher, Massachusetts, America) and analyzed by Modfit software.

### Wound healing and transwell assays

The wound healing assay was carried out to determine the migration ability of cells. Cells were digested by trypsin and seeded in six-well plates (500,000 cells per well). After confluence, cells were scratched with a 200 µl pipette tip, washed with PBS twice and cultured in serum-free medium. Wound closure was observed and photographed under a microscope at 0 h and 24 h.

The invasive ability of BLCA cells was detected by Transwell assay. A mixture of Matrigel and PBS (1:8) was added to the upper chambers of 24-well Transwell plates with an 8.0 μm aperture (Corning, NY, USA) and then maintained at 37 °C until curdling. Then, 1.0 × 10^5^ cells suspended in 100 μl serum-free medium were seeded into the upper chambers, while 700 µl medium containing 20% FBS was added to the lower chambers. After incubation for 24 h (T24 cells) or 48 h (5637 cells), cells were fixed with 4% formaldehyde and stained with 0.1% crystal violet. Five random fields of view were selected and analyzed in each chamber. Images were collected with an inverted microscope.

### Immunofluorescence (IF)

Cells were fixed with 4% paraformaldehyde for 15 min followed by 0.5% Triton X-100 for 10 min, incubated with 3% bovine serum albumin (BSA) for 1 h at room temperature, and then incubated with primary antibodies overnight at 4 °C. The next day, cells were incubated with secondary antibodies labeled with Alexa Fluor™ 555/633/488 (1:300, Proteintech) for 1 h at room temperature, and stained with DAPI (10 μg/ml) for 5 min at room temperature. Finally, the cells were covered with an anti-fluorescence-quenching sealing liquid. Images were taken under a fluorescence microscope.

### Co-immunoprecipitation (Co-IP)

The total protein of cells in each group was extracted. Protein A/G magnetic beads (HY-K0202, MedChemExpress) were washed with PBST, incubated with diluted anti-P-EGFR or control IgG antibodies for 2 h at 4 °C, washed with PBST again and incubated with total protein extracts for 2 h at 4 °C. After that, the magnetic beads were washed four times, suspended in 50 μl of 1 × loading buffer, and boiled for 5 min. The obtained protein samples were analyzed by western blot.

### Animals and xenograft model

Animal experiments followed the guidelines and approved protocols of the Ethics Committee of Tongji Hospital. Five-week-old male NCG mice were purchased from GemPharmatech (Nanjing, China) and housed in specific pathogen-free conditions for one week before experiments. Mice were randomly divided into the NC group and sh-ANXA1 group (n = 5 mice per group). NC and sh-ANXA1 5637 cells (1 × 10^6^) were subcutaneously injected into the right upper back of NC group and sh-*ANXA1* group mice, respectively. Tumor lengths and widths were measured with Vernier calipers starting on day nine and then measured every three days. Tumor volume was calculated using the following formula: V (mm^3^) = length × width^2^/2. At the end of the experiment, mice were sacrificed by injecting excessive 2% pentobarbital sodium. The xenograft tumors were completely separated, weighed and fixed with 4% paraformaldehyde for IHC.

### Statistical analyses

GraphPad 8 and SPSS 21 software were used for statistical analyses. Student’s t-test was used for the comparison of two groups. The OS of BLCA patients with different expression levels of ANXA1 was determined by Kaplan–Meier analysis. Chi-square tests or Fisher’s exact tests were conducted to investigate the clinical relevance of ANXA1 expression in BLCA. Univariate and multivariate Cox regression analyses were performed to identify risk factors for prognosis in BLCA. The relationship between ANXA1 and EGFR was evaluated by Pearson’s correlation analysis. All data were analyzed using two-tailed tests, and a p value < 0.05 was considered statistically significant. The results were shown as the mean ± SD.

## Results

### High expression level of ANXA1 predicts tumor progression and poor prognosis in BLCA

**Fig. 1:**
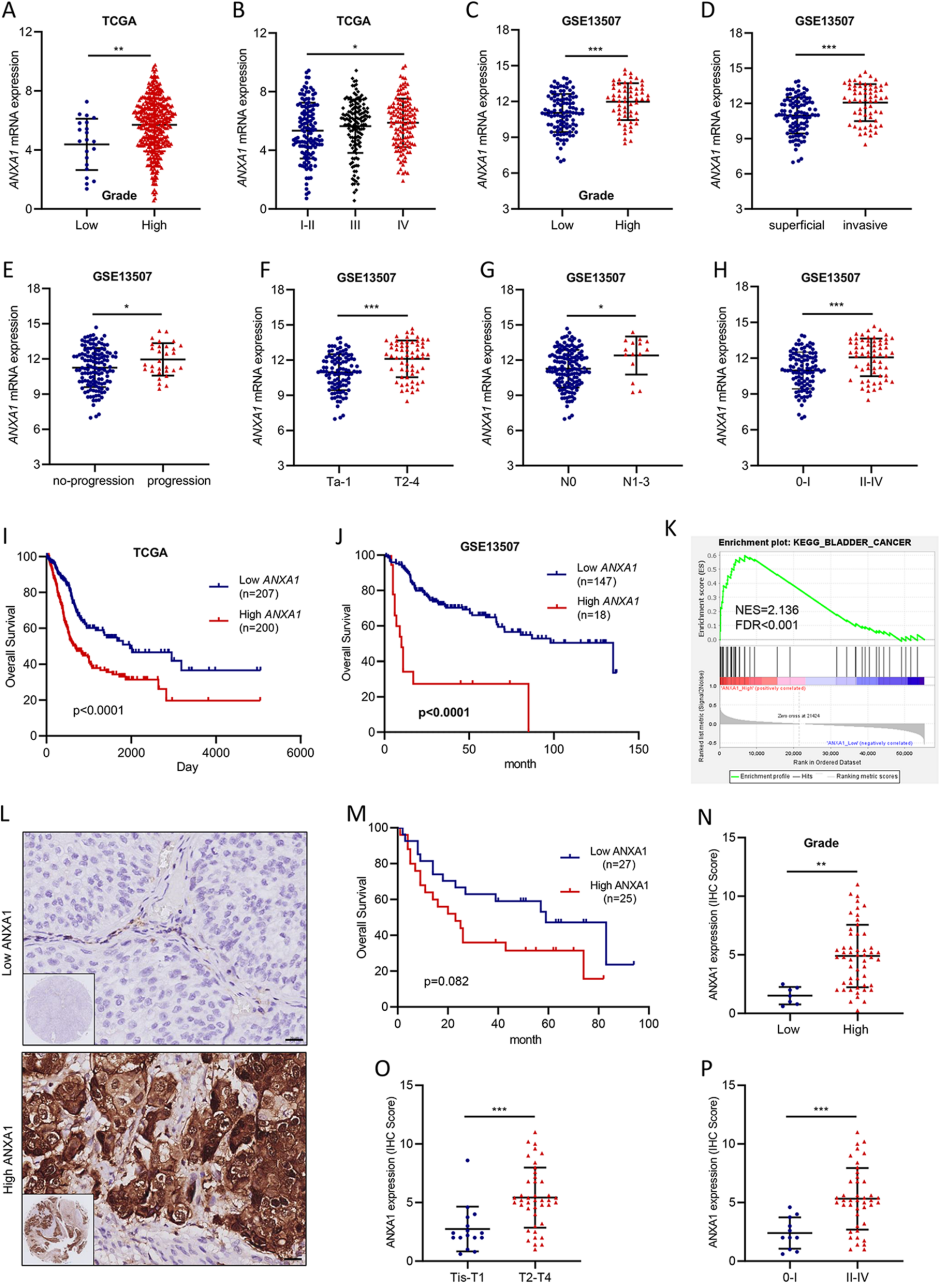
ANXA1 overexpression correlates with tumor progression and poor prognosis in human BLCA. **A**, **B** The association of *ANXA1* gene expression with clinicopathologic features in TCGA and GSE13507 datasets. **I**, **J** Kaplan–Meier curves of OS of BLCA patients in TCGA (p < 0.001) and GSE13507 datasets (p < 0.001). **K** GSEA analysis of specimens with high and low expression of ANXA1 based on the TCGA dataset (KEGG_BLADDER_CANCER, NES = 2.136, FDR < 0.001). **L** Representative high ANXA1 protein and low ANXA1 protein staining patterns in BLCA tissues (original magnification × 400) (scale bar: 20 μm). **M** Kaplan–Meier survival curve of BLCA patients with high ANXA1 and low ANXA1 expression (p = 0.082). **N**–**P** The association of ANXA1 protein expression with clinicopathologic features in BLCA samples. According to the optimal cutoff value calculated by X-tile software, samples were divided into high ANXA1 and low ANXA1 groups. *p < 0.05, ** *p* < 0.01, *** *p* < 0.001

To investigate the underlying roles of *ANXA1* in BLCA, we first analyzed the expression pattern of *ANXA1* in TCGA and GEO datasets (GSE13507). The expression of *ANXA1* was positively correlated with histological grade (Fig. 1A, C, Table 1), invasion (Fig. 1D), progression (Fig. 1E), T stage (Fig. 1F), N stage (Fig. 1G), and clinical stage (Fig. 1B, H, Table 1). In addition, based on the TCGA and GEO datasets, the Kaplan–Meier method and log-rank test were used to detect the correlation between ANXA1 expression and OS in BLCA patients. According to the optimum cutoff threshold calculated by X-tile software, the samples of the TCGA and GEO datasets were divided into high *ANXA1* (TCGA: n = 200, GEO: n = 18) and low *ANXA1* (TCGA: n = 207, GEO: n = 147) groups. Consistently, patients with high *ANXA1* had shorter OS than those with low *ANXA1* in the TCGA (Fig. 1I) and GSE13507 (Fig. 1J) datasets. In the TCGA dataset, the median OS was 1971 days in the low *ANXA1* group and 680 days in the high *ANXA1* group. OS of the low *ANXA1* group was significantly longer than that of the high *ANXA1* group (hazard ratio (HR), 1.96; 95% CI, 1.46 to 2.64; P < 0.0001). In the GEO dataset, the median OS was 135 months in the low *ANXA1* group and 10 months in the high *ANXA1* group. OS of the low *ANXA1* group was significantly longer than that of the high *ANXA1* group (HR, 4.258; 95% CI, 1.467 to 12.36; P < 0.0001). GSEA analysis demonstrated that the gene set relevant to bladder cancer was enriched in TCGA samples with high *ANXA1* expression (Fig. 1K). ROC curves were constructed to evaluate the prognostic prediction value of *ANXA1*. The results showed that *ANXA1* might have a certain ability in outcome prediction of BLCA patients but low accuracy (AUC of OS: 0.626, 1-year survival: 0.665, 2-year survival: 0.658, 5-year survival: 0.640) (Additional file 1: Fig. S1A, B). A univariate Cox regression model revealed that age, tumor depth, lymph node metastasis, distant metastasis, clinical stage, and *ANXA1* expression were associated with the prognosis of BLCA patients in terms of OS (Table 2). Multivariate analysis after adjustment indicated that *ANXA1* was an independent prognostic factor for BLCA (HR = 2.34, 95% CI: 1.403–3.909, P = 0.001) (Table 2).

**Table 1:** Correlation of *ANXA1* expression with clinicopathological factors

Parameters		*ANXA1*		*p*-value
		Low	High	
Age (years)				0.175
≤ 60	108	59	49	
> 60	304	143	161	
Gender				0.119
Male	304	156	148	
Female	108	46	62	
Histological grade				0.034*
Low	21	15	6	
High	388	185	203	
Tumor depth				0.055
T0–T2	124	67	57	
T3–T4	255	111	144	
Lymph node metastasis				0.143
N0	239	123	116	
N1–N3	131	57	74	
Distant metastasis				0.918
M0	196	110	86	
M1	11	6	5	
Clinical stage				0.008*
I + II	133	78	55	
III + IV	277	124	153	

*p < 0.05

**Table 2:** Univariate and Multivariate Cox proportional hazards analysis of OS

Variable	Univariate			Multivariate		
	HR	95%CI	*p*-value	HR	95%CI	*p*-value
Age (> 60/ ≤ 60)	1.851	1.455–2.675	0.002*	1.524	0.808–2.876	0.193
Gender (Female/Male)	1.107	0.789–1.535	0.543			
Grade (High/Low)	2.867	0.709–11.588	0.139			
Tumor depth (T3–T4/T0–T2)	2.203	1.509–3.217	< 0.001*	2.597	0.841–8.023	0.097
Lymph node (N1–N3/N0)	2.359	1.723–3.230	< 0.001*	1.711	0.972–3.012	0.063
Distant metastasis (M1/M0)	3.306	1.580–6.917	0.002*	1.63	0.615–4.325	0.326
Clinical stage (III + IV/I + II)	2.288	1.573–3.328	< 0.001*	1.51	0.443–5.146	0.51
*ANXA1* (High/Low)	1.973	1.455–2.675	< 0.001*	2.34	1.403–3.909	0.001*

^*^p < 0.05

To further validate the roles of ANXA1 in BLCA, BLCA tissue samples, including 63 cancer tissues and 16 normal tissues adjacent to cancer, were subjected to immunohistochemistry (IHC) staining (Fig. 1L). There was no significant difference in the expression level of ANXA1 between tumor tissue and adjacent normal tissue of 16 BLCA samples (Additional file 1: Fig. S1C). Fifty-Two patients with detailed survival information were divided into the low ANXA1 group (n = 27) and high ANXA group (n = 25). Although there was no statistically significant difference in OS between the two groups, OS of the low ANXA1was longer group than that of the high ANXA1 group (HR for death, 1.978; 95% CI, 0.9593 to 4.080; P = 0.082) (Fig. 1M). The median overall survival was 59 months in the low ANXA1 group and 23 months in the high ANXA1 group. In addition, up-regulation of ANXA1 was observed in advanced patients and patients with high histological grade (Fig. 1N–P, Additional file 1: Fig. S1D, E). All these observations demonstrated that elevated expression of ANXA1 was significantly related to the progression and poor prognosis of BLCA.

### Knockdown of *ANXA1* inhibits BLCA cell proliferation in vitro

**Fig. 2:**
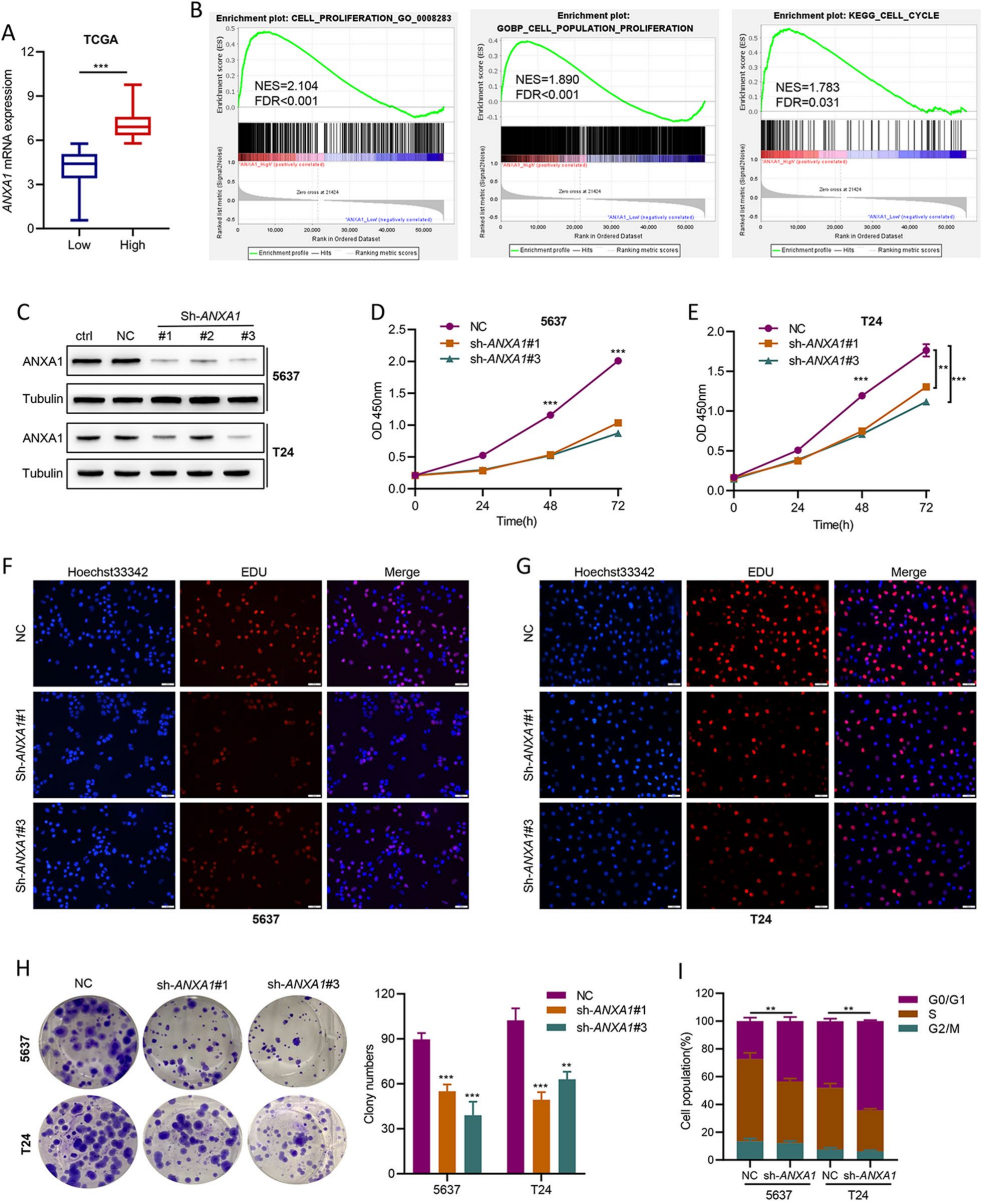
*ANXA1* knockdown impairs proliferation, colony formation, and the G1-to-S phase transition in BLCA cell lines. **A** *ANXA1* expression levels in high *ANXA1* samples compared with their low *ANXA1* counterparts among 414 BLCA samples in the TCGA cohort. The optimal cutoff value of low and high ANXA1 was calculated by X-tile software. **B** GSEA analysis of proliferation and cell cycle target gene sets based on the TCGA cohort. **C** Western blot of ANXA1 in BLCA cell lines transfected with negative control sh-RNA and three different sh-RNAs specific to *ANXA1*. Ctrl: normal cells; NC: cells transfected negative control sh-RNA; sh-*ANXA1*#1–3: cells transfected three different sh-RNAs specific to *ANXA1*. **D**, **E** A CCK-8 assay was conducted to detect the effect of ANXA1 on cell viability. **F**, **G** DNA synthesis in BLCA cells of the NC and sh-*ANXA1* groups was examined by EdU assay. **H** Colony formation assay of NC and sh-*ANXA1* groups. **I** Cell cycle assay of NC and sh-*ANXA1* groups. *p < 0.05, ** *p* < 0.01, *** *p* < 0.001

To clarify the biological functions of ANXA1 in BLCA, we conducted bioinformatics analysis based on the TCGA dataset and a series of in vitro cell experiments. The samples in the TCGA dataset were divided into low *ANXA1* and high *ANXA1* groups based on the optimum cutoff threshold calculated by X-tile software (Fig. 2A). GSEA demonstrated that gene sets relevant to the proliferation and cell cycle were enriched in the high *ANXA1* group (Fig. 2B). Furthermore, BLCA cell lines (5637 and T24) with *ANXA1* knockdown were constructed using gene-specific shRNA. Western blotting and qRT–PCR were performed to confirm *ANXA1* knockdown in the constructed cell lines (Fig. 2C, Additional file 2: Fig. S2A, B). The results of CCK-8 and EdU assays indicated that loss of *ANXA1* attenuated the proliferation of BLCA cells (Fig. 2D–G). Additionally, colony formation was significantly suppressed after *ANXA1* knockdown (Fig. 2H). Flow cytometric analysis revealed that loss of *ANXA1* profoundly increased the G0/G1 phase cell fraction and decreased the S phase cell fraction, which demonstrated that knockdown of *ANXA1* might suppress the G1-to-S phase transition in BLCA cells (Fig. 2I, Additional file 2: Fig. S2C).

### Knockdown of *ANXA1* suppresses BLCA cell migration, invasion and EMT in vitro

**Fig. 3:**
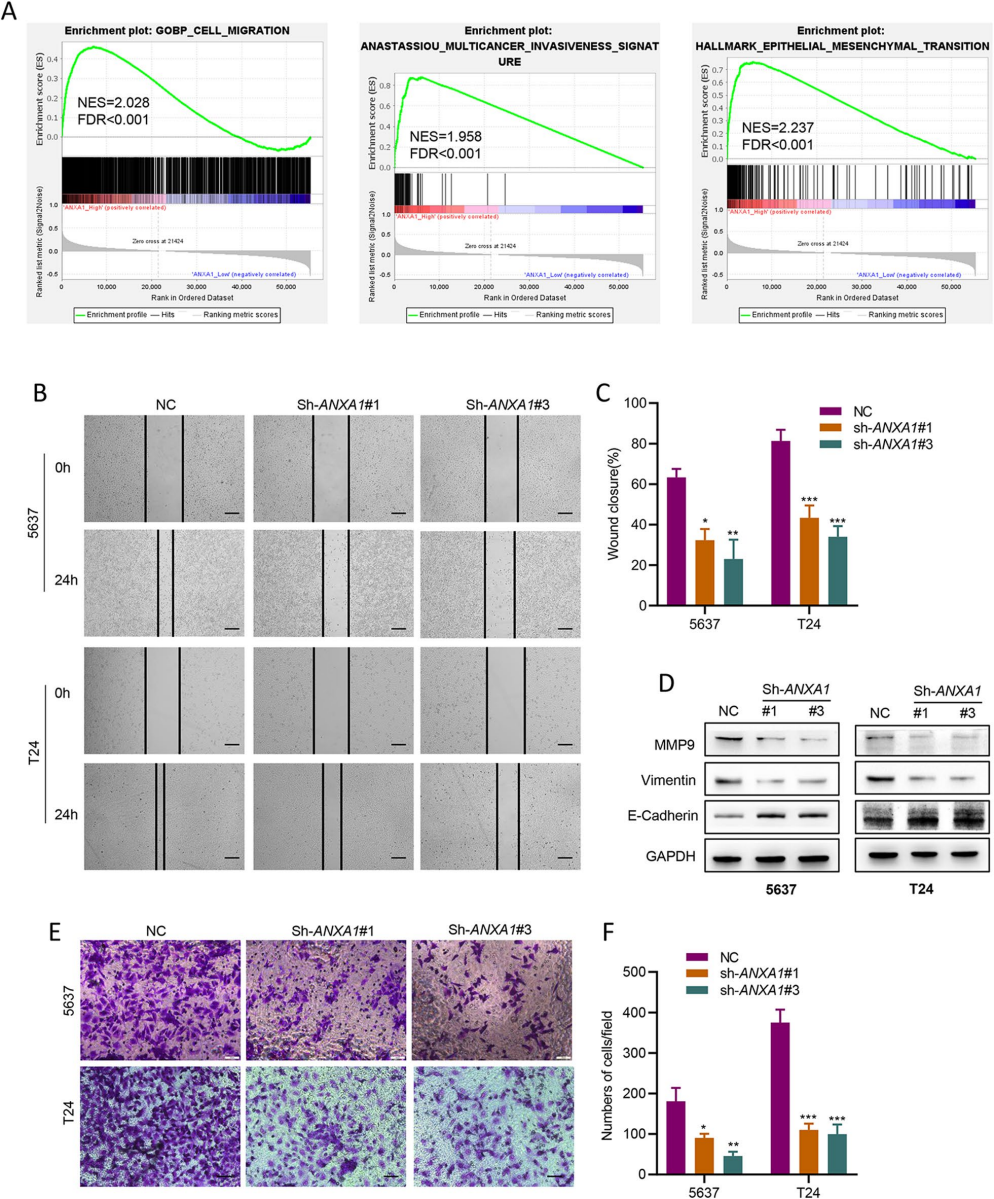
Loss of *ANXA1* suppresses the migration, invasion, and EMT of BLCA cells. **A** GSEA analysis of migration, invasion and EMT in high and low *ANXA1* samples based on the TCGA dataset. **B**, **C** The migration ability of BLCA cells was detected by wound healing assays (scale bar:50 μm). **D** The expression of MMP9, E-cadherin and vimentin was evaluated by western blot in BLCA cell-transfected lentiviruses. **E**, **F** The invasion ability of BLCA cells was detected by Transwell assay (scale bar: 50 μm). *p < 0.05, ** *p* < 0.01, *** *p* < 0.001

Given our previous finding that ANXA1 is associated with the clinical progression of BLCA, we sought to investigate whether ANXA1 is capable of facilitating invasion and migration in BLCA. GSEA analysis based on the TCGA cohort indicated that high expression of *ANXA1* was strongly linked with migration, invasion and epithelial–mesenchymal transition (EMT) (Fig. 3A). Then, the results of wound healing and Transwell assays verified that the migration and invasion abilities of BLCA cells decreased after *ANXA1* knockdown (Fig. 2B, C, E, F). We further detected the levels of the EMT markers E-cadherin, vimentin and matrix metalloproteinase 9 (MMP9) by western blotting. Consistently, the results indicated that knockdown of *ANXA1* repressed MMP9 and Vimentin, and elevated E-cadherin (Fig. 3D). Collectively, these findings implied that silencing *ANXA1* suppressed the migration and invasion capacities of BLCA cells.

### The relationship between ANXA1 status and EGFR signaling in BLCA

**Fig. 4:**
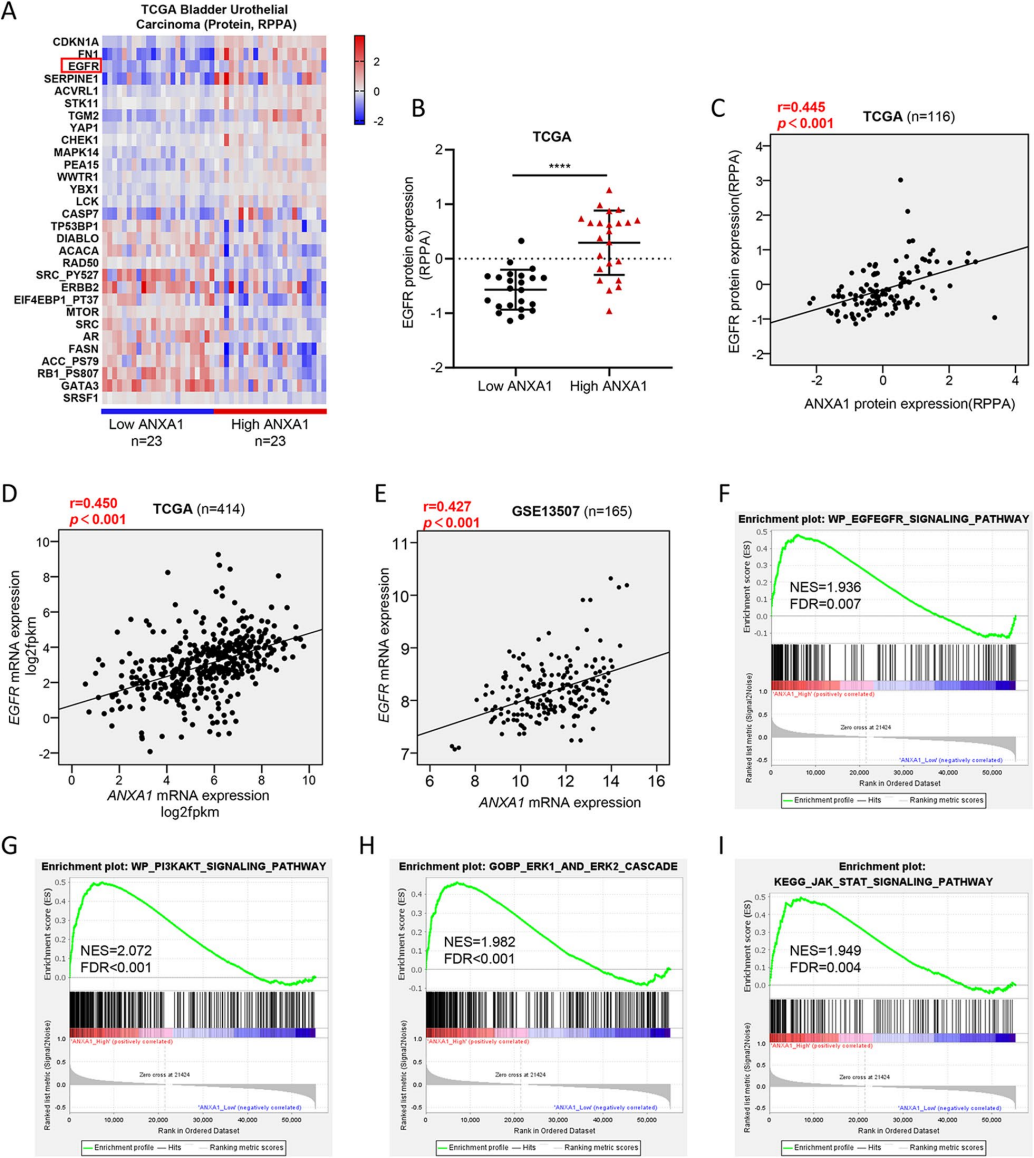
Bioinformatics analysis based on TCGA and GEO datasets reveals a link between ANXA1 and EGFR signaling in BLCA. **A** Heatmaps showing 30 proteins significantly differentially expressed in the high ANXA1 and low ANXA1 protein expression groups from the TCGA dataset. **B** Individual value plot showing the protein expression of EGFR between the high and low ANXA1 groups in the TCGA dataset. **C** Pearson correlation analysis of ANXA1 and EGFR protein expression (RPPA) in the TCGA dataset. **D**, **E** Pearson correlation analysis of ANXA1 and EGFR gene expression in TCGA and GEO datasets. **F**–**I** GSEA analysis of EGF/EGFR, PI3K/AKT, ERK and JAK/STAT signaling in high and low ANXA1 samples based on the TCGA dataset

To gain insight into the molecular mechanism of ANXA1 as a cancer promoting factor, we conducted in-depth analyses of the TCGA dataset. Protein (RPPA) and Gene, RNASeq (IlluminaHiSeq) datasets of the TCGA bladder cancer cohort were downloaded from cBioPortal for Cancer Genomics Browser. According to the ANXA1 protein expression level, the samples with the highest 20% and lowest 20% ANXA1 were regarded as the low ANXA1 and high ANXA1 groups. The unpaired t-test was used to screen the differentially expressed proteins between the high ANXA1 and low ANXA1 groups. A heatmap of thirty differentially expressed proteins between the two groups, including EGFR, is shown in Fig. 4A. The scatter dot plot and waterfall plot displayed the EGFR expression of each sample in two groups (Fig. 4B, Additional file 3: Fig. S3A). Moreover, Pearson correlation analysis and linear regression analysis further demonstrated that the protein expression level of EGFR was significantly correlated with that of ANXA1 in 116 BLCA samples (r = 0.445, p < 0.001) (Fig. 4C). Consistently, the expression level of *EGFR* was positively correlated with that of *ANXA1* in the TCGA, GSE13507 and GSE19915 datasets (Fig. 4D, E, Additional file 3: Fig. S3B, C). In addition, *ANXA1* was significantly positively correlated with *EGFR* in different clinical stages in the TCGA dataset (Additional file 3: Fig. S3D–F).

To further demonstrate that ANXA1 was involved in regulating EGFR signaling, we performed GSEA analyses and found that gene sets relevant to activation of EGFR signaling were enriched in samples with high *ANXA1* expression (Fig. 4F, Additional file 3: Fig. S3G). Moreover, increased expression of ANXA1 was closely related to activation of the PI3K/AKT, MEK/ERK and JAK/STAT3 pathways, which are critical downstream pathways of EGFR signaling in regulating cellular functions, including survival, proliferation, differentiation, and motility (Fig. 4G–I). These results suggested that ANXA1 might interact with the EGFR signaling pathway.

### ANXA1 regulates EGFR signaling and the downstream pathways in vitro

**Fig. 5:**
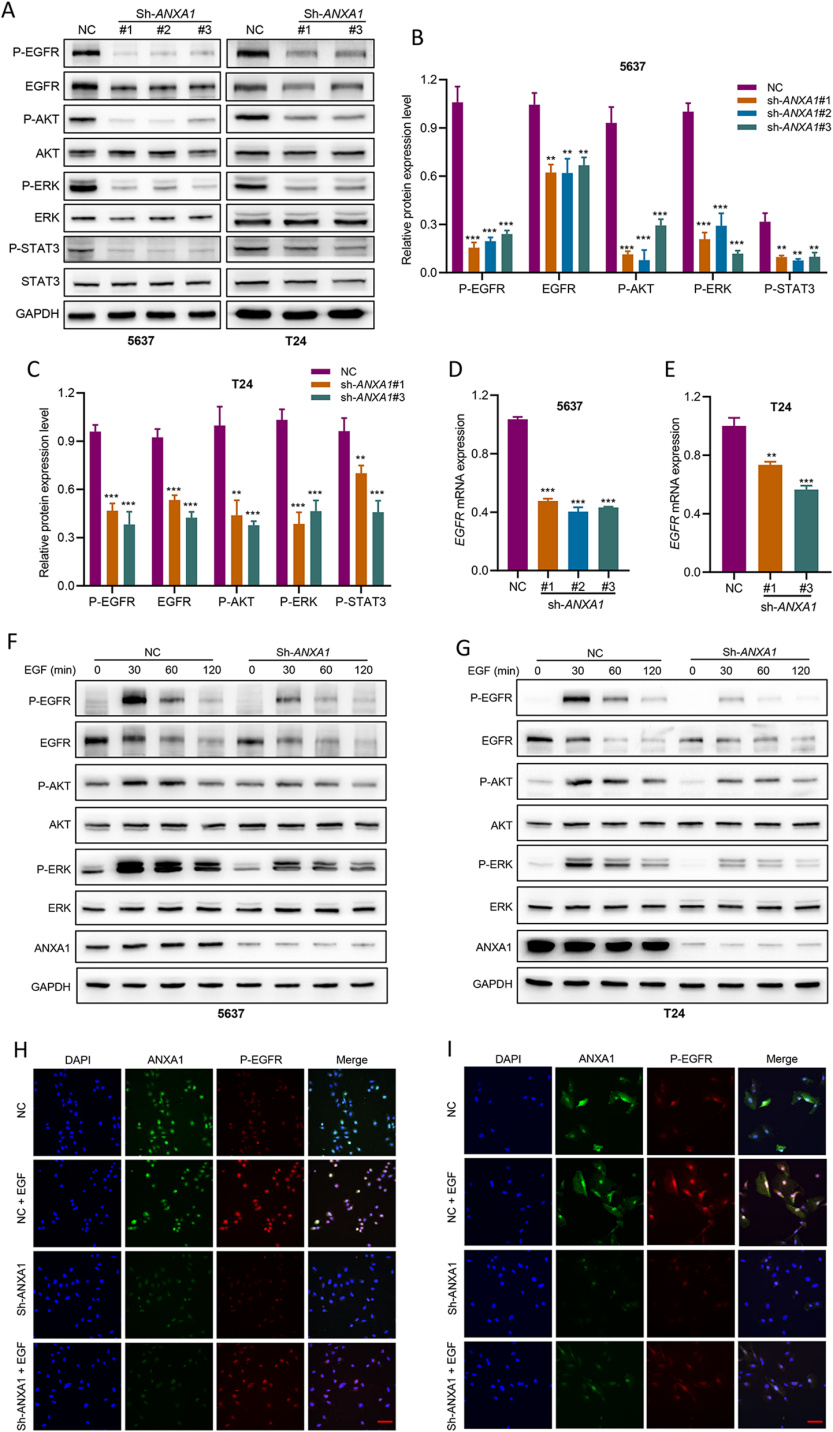
*ANXA1* knockdown inhibits the expression and phosphorylation of EGFR and the activation of downstream pathways. **A**–**C** The protein levels of EGFR, p-EGFR (Tyr1068), AKT, p-AKT (Ser473), ERK, and p-ERK (Thr202/Tyr204) in BLCA cells with or without *ANXA1* knockdown were measured by Western blot. GAPDH was used as an internal reference. **D**, **E** The mRNA levels of EGFR in BLCA cells with or without *ANXA1* knockdown were measured by qRT–PCR. **F**, **G** BLCA cells were treated with EGF (100 ng/ml) for different durations. The levels of EGFR, p-EGFR (Tyr1068), AKT, p-AKT (Ser473), ERK, and p-ERK (Thr202/Tyr204) were measured by Western blot. **H**, **I** Co-localization of p-EGFR and ANXA1 in the NC and sh-*ANXA1* groups was detected by immunofluorescence staining (scale bar: 50 μm)

EGFR signaling plays important roles in tumorigenesis and progression [30]. We investigated the effect of ANXA1 on the regulation of EGFR signaling in BLCA cells. A substantial reduction in the gene and protein expression of EGFR was detected in ANXA1 knockdown cells by qRT–PCR and western blot (Fig. 5A–E). The expression levels of major proteins related to the EGFR signaling cascade were detected in BLCA cells transfected with lentivirus. As observed in Fig. 5A, *ANXA1* knockdown markedly decreased the phosphorylation of EGFR(Tyr1068). In addition, the key downstream regulators of EGFR signaling, including phosphorylated AKT (P-AKT), phosphorylated ERK (P-ERK), and phosphorylated STAT3 (P-STAT3), were significantly reduced after depletion of *ANXA1* (Fig. 5A).

**Fig. 6:**
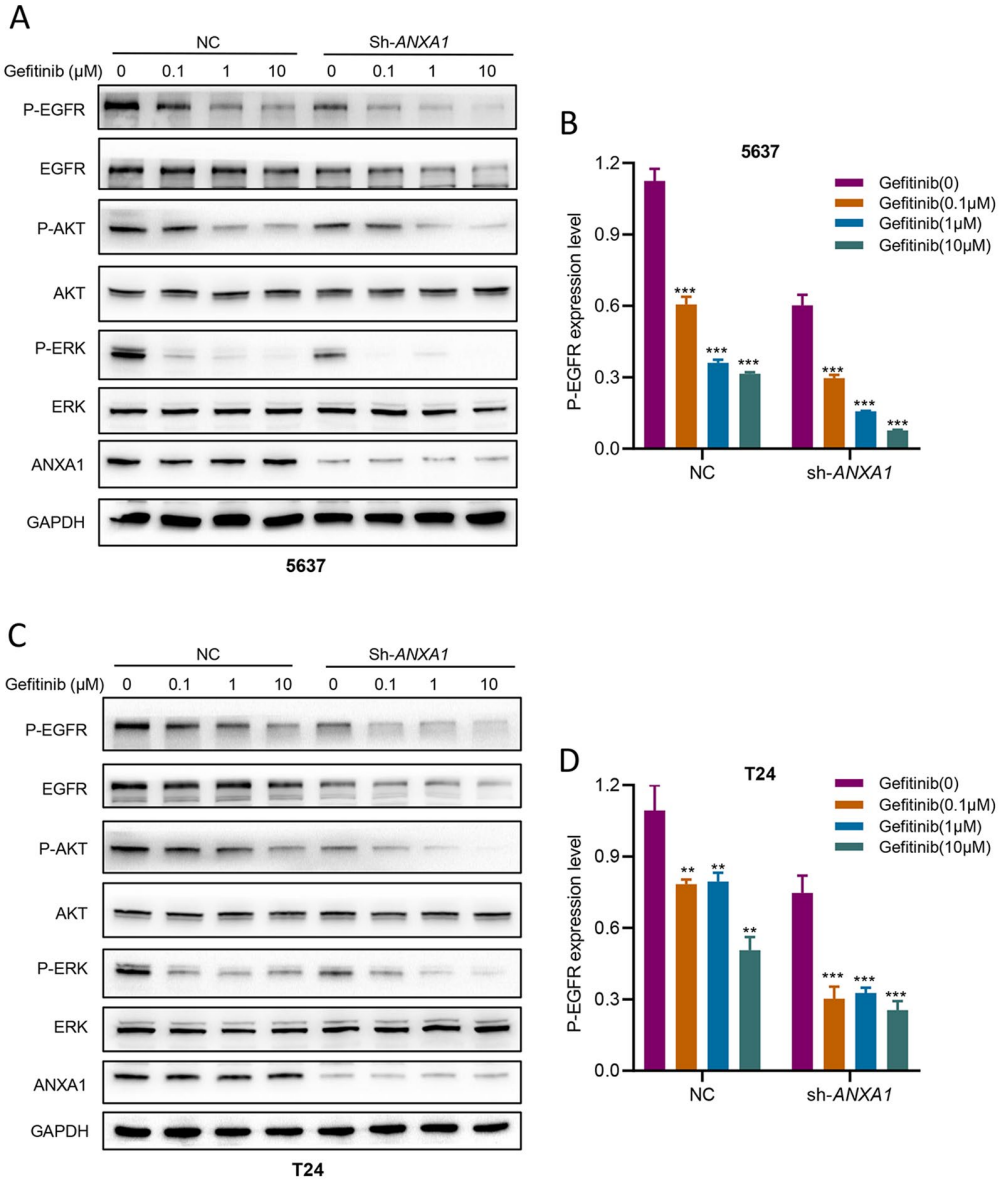
*ANXA1* knockdown promoted the inactivation of EGFR signaling and downstream pathways after treatment with gefitinib. **A**, **C** BLCA cells were treated with different concentrations of gefitinib (0.1 μM, 1 μM, 10 μM) for 6 h. The levels of EGFR, p-EGFR (Tyr1068), AKT, p-AKT (Ser473), ERK, and p-ERK (Thr202/Tyr204) were measured by Western blot. **B**, **D** Densitometric ratios of P-EGFR/GAPDH. Data are presented as the mean ± SD

We further explored the effect of ANXA1 on EGFR signaling in response to EGF and gefitinib. The results demonstrated that loss of *ANXA1* attenuated the activation of EGFR after treatment with EGF (Fig. 5F–I). Similarly, knockdown of *ANXA1* significantly reduced EGF-induced phosphorylation levels of the downstream mediators ERK, STAT3 and AKT (Fig. 5F, G). In addition, loss of *ANXA1* promoted the inactivation of EGFR signaling and downstream pathways after treatment with gefitinib (Fig. 6A–D). Together, our results indicated that ANXA1 regulated the activation of EGFR and its downstream pathways.

### Loss of ANXA1 promotes P-EGFR ubiquitination and degradation

**Fig. 7:**
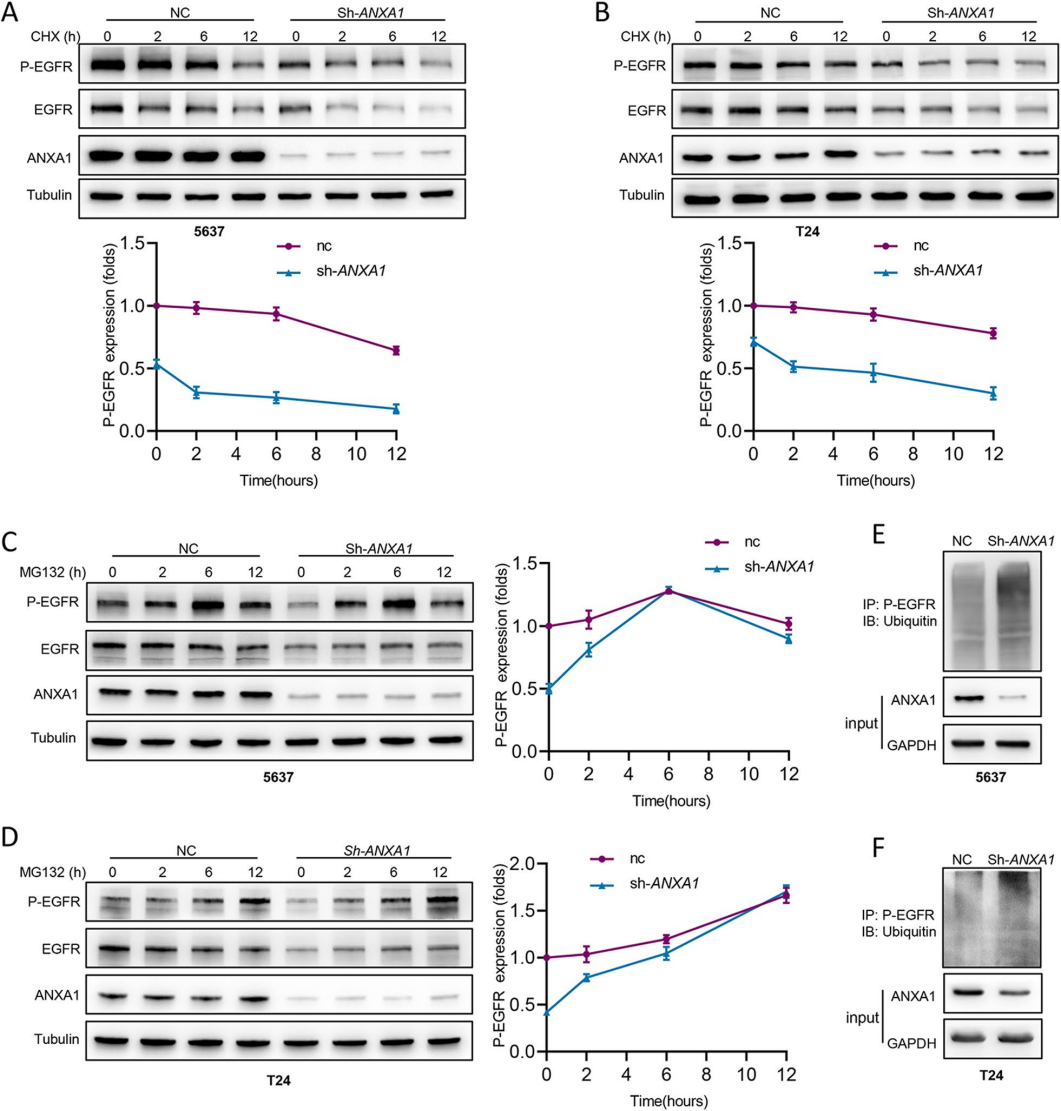
*ANXA1* knockdown promotes p-EGFR ubiquitination and degradation. **A**, **B** After treatment with CHX(20 μM) for different durations, the levels of EGFR and P-EGFR in the NC and sh-*ANXA1* groups were detected by Western blot. **C**, **D** After treatment with MG132(10 μM) for different durations, the levels of EGFR and p-EGFR in the NC and sh-*ANXA1* groups were detected by Western blotting. **E**, **F** At 6 h post-treatment with MG132 (10 μM), cell lysates were obtained and immunoprecipitated with an anti-p-EGFR antibody, followed by immunoblotting against ubiquitin. GAPDH was used as an internal reference

To gain insight into the mechanism of ANXA1-mediated EGFR signaling in BLCA cells, we evaluated whether ANXA1 regulated EGFR signaling directly or indirectly. ANXA1 could not directly bind to EGFR in BLCA cells (data not shown). Then, the half-lives of EGFR and P-EGFR were detected after the cells were treated with cycloheximide (CHX, 20 μM) to block protein synthesis. Notably, the stability of P-EGFR was significantly decreased in BLCA cells with *ANXA1* knockdown. At the same time, there was no observable difference in the stability of EGFR between NC cells and *ANXA1* knockdown cells. In addition, *ANXA1* silencing inhibited the gene expression of EGFR (Fig. 5D, E). These results suggested that ANXA1 might regulate EGFR signaling by promoting the expression of EGFR and maintaining the stability of P-EGFR (Fig. 7A, B). Furthermore, approximately 6 h after MG132 (10 μM) treatment in 5637 cells and 12 h after MG132 (10 μM) treatment in T24 cells, P-EGFR reached similar levels in both groups (Fig. 7C, D). To investigate whether ANXA1 could regulate ubiquitination of P-EGFR, after treatment with MG132 (10 μM) for 6 h, cell lysates were immunoprecipitated with anti-P-EGFR antibody, followed by western blotting of ubiquitin. Compared with NC cells, the ubiquitination level of P-EGFR was higher in cells with *ANXA1* knockdown (Fig. 7E, F).

### ANXA1 knockdown suppresses tumor growth in human BLCA xenograft mice

**Fig. 8:**
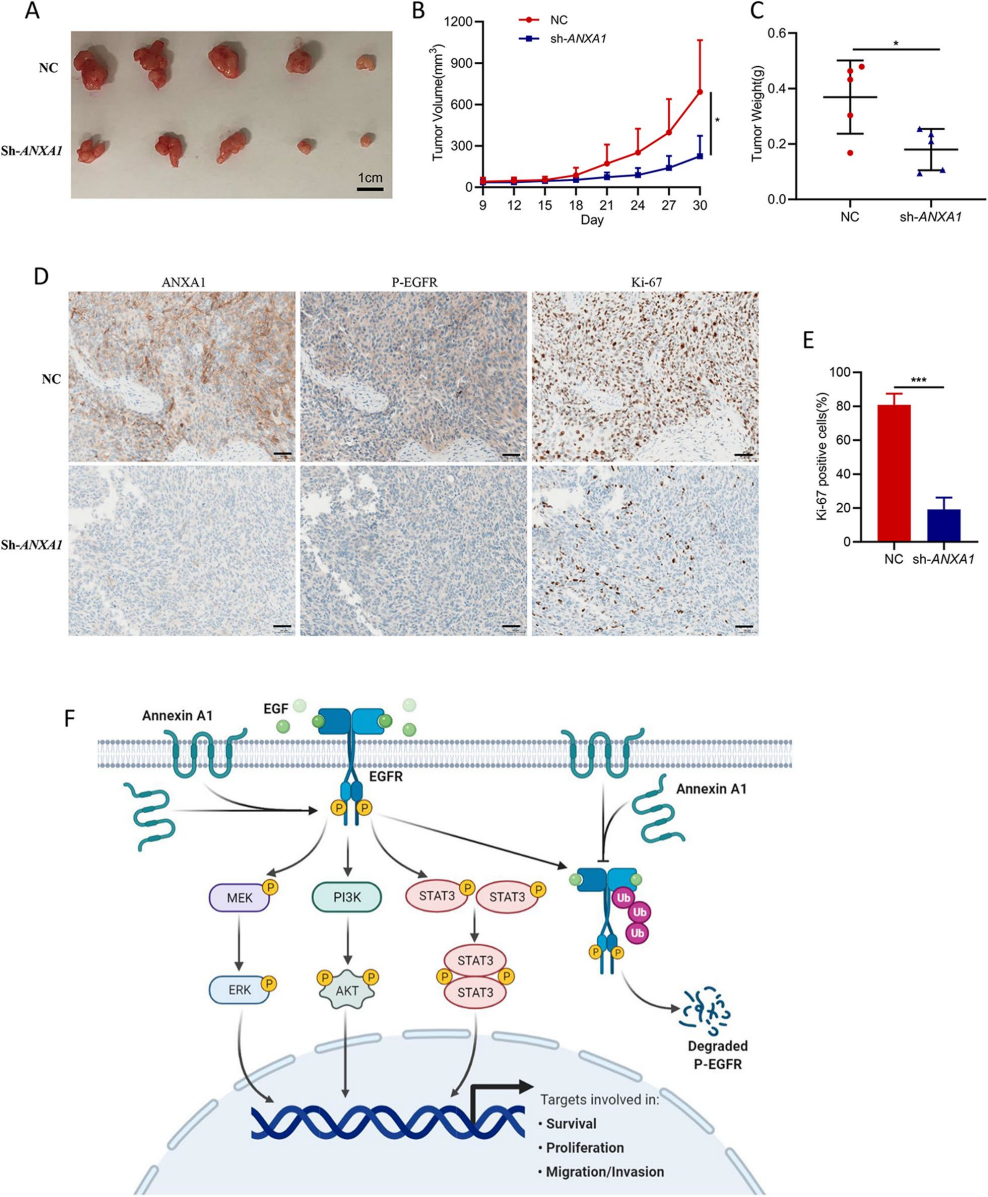
Knockdown of *ANXA1* inhibits tumor growth in subcutaneous xenograft models. **A** Gross appearance of tumors from NCG mice injected with 5637 NC or 5637 sh-*ANXA1* cells. **B** Tumor growth was monitored for 30 days. The tumor volume of each group is shown as a line chart. **C** Individual value plot shows the tumor weights of the NC and sh-*ANXA1* groups. **D** Representative IHC staining patterns of ANXA1, P-EGFR and Ki-67 in each group are shown (original magnification 200 ×) (scale bar: 50 μm). **E** Quantification of the number of Ki-67 positive cells in each group. **F** Proposed possible mechanism of ANXA1 functions in BLCA. ANXA1 promotes EGFR expression and inhibits P-EGFR degradation to activate EGFR signaling and its downstream pathways, thereby facilitating the proliferation, invasion and migration of BLCA cells

We sought to determine whether ANXA1 could affect tumor growth in vivo. Stabilized *ANXA1* knockdown 5637 cells were subcutaneously implanted into NCG mice (1 × 10^6^ cells per mouse, five mice per group). The mice injected with the corresponding NC cells served as a control group. As expected, the results indicated that the tumor volume and weight of the *ANXA1* knockdown group were significantly smaller than those of the control group (Fig. 8A–C). IHC staining showed that P-EGFR and Ki-67 expression significantly decreased in tumor tissues of the *ANXA1* knockdown group (Fig. 8D, E). These results were consistent with the in vitro experiments, suggesting that ANXA1 might promote tumor growth mediated by EGFR signaling activation.

## Discussion

Bladder cancer is one of the most frequent cancers worldwide. Despite continuous therapeutic advances in other cancers, there have been few advances in the treatment of bladder cancer over the past three decades [7]. This trend has considerably changed in the past few years. An improved understanding of the genetics and molecular biology of BLCA has emphasized the importance of screening better predictive biomarkers and novel therapeutic targets [2, 8]. Herein, we found that ANXA1 might be an effective prognostic predictor and therapeutic target for BLCA. We further conducted an in-depth exploration of the expression pattern, biological functions, and potential mechanism of ANXA1 in the development of BLCA. Our study indicated that ANXA1 facilitated the proliferation, invasion and migration of BLCA cells by activating the EGFR signaling cascade, which ultimately led to the progression and poor prognosis of BLCA (Fig. 8F). Although ANXA1 was initially discovered due to its role in inflammation, accumulated evidence has subsequently shown that dysregulation of ANXA1 is strongly linked with tumorigenesis and the development of some cancer types [14]. Upregulation of ANXA1 has been observed in lung cancer, pancreatic cancer, colorectal cancer, and melanomas, and has a notable correlation with advanced stages and unfavorable prognosis [19, 23, 31–33]. Previous studies suggested that ANXA1 was up-regulated in BLCA tissues, related to high histological grade and escalated T status, and predicted disease-specific survival and metastasis-free survival [25–27]. Based on these studies, we conducted a comprehensive study to explore the expression pattern of ANXA1 in BLCA through bioinformatics analysis of two independent datasets and IHC staining of BLCA tissue samples. The results confirmed that a high expression level of ANXA1 was markedly related to poor differentiation, tumor stage and unfavorable prognosis of bladder cancer. However, there was no significant difference in the expression level of ANXA1 between tumor tissue and adjacent normal tissue of BLCA samples. Only 16 normal tissues of BLCA patients were included in this study. The patient’s clinical stage and treatment were not uniform. It is necessary to expand the sample size and formulate unified inclusion standards to reduce the influence of these factors on the experimental results. Furthermore, univariate and multivariate Cox analyses indicated that ANXA1 was a prognostic factor for BLCA. All of these results suggested that high expression of ANXA1 might contribute to the development and progression of BLCA and be a risk factor for the clinical outcome of BLCA.

Numerous investigations have revealed that ANXA1 is involved in tumor proliferation, invasion, migration and EMT in vitro and in vivo. For instance, ANXA1 has been proven to promote the proliferation of esophageal squamous carcinoma cells and breast cancer cells and facilitate EMT to enhance the migration and invasion of metastatic breast cancer cells [34–36]. Ming Yi et al. reported that tumor growth and metastasis were observably inhibited in *anxa1* knockout mice [37]. To date, there is no relevant research on the biological functions of ANXA1 in BLCA cells. Here, we conducted a series of in vivo and in vitro experiments to study the biological functions of ANXA1 in BLCA cells. Knockdown of *ANXA1* promoted cell cycle arrest by increasing the G0/G1 phase cell fraction and decreasing the S phase cell fraction. One of the defining features of cellular senescence is cell cycle arrest. Despite the general consensus that senescence plays a primarily and immediately tumor-suppressive role in many settings, the long-term implications of senescent cells are potentially detrimental [38–41]. These results indicated that silencing *ANXA1* inhibited the proliferation of BLCA cells in vitro and the growth of xenograft tumors in vivo. Moreover, loss of *ANXA1* suppressed the migration and invasion abilities and EMT of BLCA cells. In summary, ANXA1 promoted the growth and metastasis of BLCA cells.

Epidermal growth factor receptor (EGFR), as a receptor for epidermal growth factor (EGF) and transforming growth factor-α (TGF-α), has been found to facilitate the growth and aggressiveness of cancer cells [30]. Phosphorylated EGFR gives rise to the activation of downstream signaling, such as the PI3K/Akt, MEK/ERK and JAK/STAT3 pathways, thereby regulating cell proliferation, migration, and differentiation [30, 42, 43]. Upregulation of EGFR has been demonstrated in many cancer types [44–46]. Likewise, EGFR is overexpressed in most patients with BLCA and unfavorable to the prognosis of patients [47, 48]. We also confirmed that BLCA patients with high *EGFR* expression had a poorer OS than those with low *EGFR* expression in the TCGA cohort (Additional file 3: Fig. S3H).

Previous studies have demonstrated that ANXA1 is linked to EGFR signaling [49–51]. In this study, we found that the protein expression level of EGFR was significantly different between the high and low ANXA1 groups in the TCGA cohort. Pearson correlation analysis between ANXA1 and EGFR was performed in three independent datasets, and the results confirmed that ANXA1 was significantly positively correlated with EGFR at the gene and protein expression levels. In addition, analysis of the combined effects of ANXA1 and EGFR showed that *ANXA1*–*EGFR*-high patients exhibited the worst overall survival rates, while patients with *ANXA1*–*EGFR* -low expression lived longest (Additional file 3: Fig. S3I). GSEA showed that gene sets related to EGFR signaling and downstream PI3K/AKT, MEK/ERK and JAK/STAT3 pathways were enriched in the high ANXA1 group. Based on previous studies and our preliminary results, we hypothesized that ANXA1 might promote tumorigenesis and development by activating the EGFR signaling cascade in BLCA. We performed further studies to validate our hypothesis and explore the relevant mechanism. After knockdown of ANXA1 in BLCA cells, EGFR signaling was significantly suppressed under both EGF-dependent and EGF-independent conditions, as well as downstream PI3K/AKT, MEK/ERK and JAK/STAT3 pathways. In-depth research found that ANXA1 maintained activation of the EGFR signaling cascade through de ubiquitination of P-EGFR in BLAC cells.

EGFR is a well-established drug target. EGFR inhibitors such as monoclonal antibodies or small molecule TKIs have been approved for the therapy of non-small cell lung cancer, head and neck squamous cell carcinoma, and colorectal cancers [52–54]. However, these drugs are effective in only a subset of cancer patients. Although EGFR inhibitors have limited efficacy in BLCA clinical trials, screening patients based on molecular profiles may help improve the efficacy of anti-EGFR treatment [55]. A study indicated that the basal-like bladder cancer subgroup is sensitive to anti-EGFR therapy [56]. In recent years, accumulated studies have suggested that molecular subtypes of BLCA are of great significance in therapeutic options [57, 58]. Due to the high biodiversity of BLCA, the development of therapies based on molecular subtypes remains challenging. Considering its role in regulating EGFR signaling, ANXA1 may be a novel therapeutic target, and targeting ANXA1 combined with EGFR inhibitors may be a new treatment strategy for BLCA patients with high ANXA1 expression. Future studies will explore the effect of ANXA1-mediated activation of EGFR signaling on the efficacy of anti-EGFR therapy for BLCA. Our study may help to screen a specific subset of BLCA patients who may benefit from treatment with EGFR inhibitors.

There are some limitations of the present study. First, the patients included in the tissue array were retrospectively analyzed, and the sample size was small, especially since only 16 adjacent normal tissues were included in the tissue array. Second, due to the high expression of ANXA1 in 5637 and T24 cells, we performed *ANXA1* knockdown experiments and further cellular experiments. The *ANXA1* overexpression experiment was not performed in this study. Finally, although tumor growth was reduced by knockdown of *ANXA1*, it did not decrease to a marginal level. There are mainly several reasons as follows. In our study, *ANXA1* was knocked down rather than completely knocked out. Compared to the NC cells, although ANXA1 was significantly reduced in BLCA cells with *ANXA1* knockdown, it was still expressed. Moreover, BLCA cells with *ANXA1* knockdown may have other compensatory mechanisms to counter tumor suppression. These limitations will be a part of our future research.

## Conclusions

In summary, our study demonstrated that high ANXA1 expression was significantly associated with the progression and poor prognosis of BLCA patients. In vitro and in vivo experiments indicated that loss of ANXA1 inhibited the growth and metastasis of BLCA cells. Further assays revealed that ANXA1 activated EGFR signaling and downstream PI3K/AKT, MEK/ERK, and JAK/STAT pathways by inhibiting P-EGFR ubiquitination and degradation. Our study suggested that ANXA1 might be an underlying prognostic biomarker and potential therapeutic target of BLCA.

## Supplementary Information

**Additional file 1. Figure S1.** (A) The ROC curves for the signature in predicting survival at time points of 1-, 2-, and 5-year in the TCGA dataset. (B) The ROC curves comparing the prognostic values of risk score and several clinical factors in the TCGA dataset. (C) The expression level of ANXA1 in tumor tissues and adjacent normal tissues of 17 patients with bladder cancer. (D) Representative ANXA1 expression in patients with different histological grades. (Original magnification ×200). (E) Representative ANXA1 expression in patients with different clinical stages. (Original magnification ×200).

**Additional file 2. Figure S2.** (A-B) qRT-PCR analysis of *ANXA1* in BLCA cell lines transfected negative control shRNA and three different sh-RNAs specific to *ANXA1*. (C) Cell cycle assay in the NC and sh-*ANXA1* groups.

**Additional file 3. Figure S3.** (A) The waterfall plot displayed the EGFR expression of each sample in the low and high ANXA1 groups. (B-F) Pearson correlation analysis of ANXA1 and EGFR gene expression in TCGA and GEO datasets. (G) GSEA analysis of EGF/EGFR signaling in high and low ANXA1 samples based on the TCGA dataset. (H) Kaplan–Meier survival curves of BLCA patients with high EGFR and low EGFR expression in the TCGA cohort. (I) Kaplan-Meier curve for the prognosis of patients with high and low ANXA1 or EGFR expression levels in the TCGA cohort.

**Additional file 4. Table S1.** Primer sequences for qRT-PCR.

## Data Availability

The data for bioinformatics analysis of this study are publicly available in The Cancer Genome Atlas (TCGA) data portal (https://tcga-data.nci.nih.gov/tcga/), Gene Expression Omnibus (GEO) database (https://www.ncbi.nlm.nih.gov/geo/), and cBioPortal for Cancer Genomics (http://www.cbioportal.org/). The rest of the data are available from the corresponding author upon reasonable request.

## Abbreviations

BLCA: Bladder cancer
EGFR: Epidermal growth factor receptor
ANXA1: Annexin A1
TCGA: The Cancer Genome Atlas
GEO: Gene Expression Omnibus
OS: Overall survival
HR: Hazard ratio
EMT: Epithelial–mesenchymal transition
NMIBC: Non-muscle-invasive bladder cancer
MIBC: Muscle-invasive bladder cancer
GSEA: Gene Set Enrichment Analysis
NES: Normalized enrichment score
IHC: Immunohistochemistry
EGF: Epidermal growth factor
NC: Negative control
PVDF: Polyvinylidene fluoride
PI: Propidium iodide
MMP9: Matrix metalloproteinases 9
TGF-α: Transforming growth factor-α
