# Correction: Annexin A1 promotes the progression of bladder cancer via regulating EGFR signaling pathway

**DOI:** 10.1186/s12935-026-04222-5

**Published:** 2026-03-06

**Authors:** Piao Li, Lingling Li, Zhou Li, Shennan Wang, Ruichao Li, Weiheng Zhao, Yanqi Feng, Shanshan Huang, Lu Li, Hong Qiu, Shu Xia

**Affiliations:** 1 Department of Oncology, Tongji Hospital, Tongji Medical College of Huazhong University of Science and Technology, 1095 Jie Fang Avenue, Hubei, 430030 Wuhan People’s Republic of China; 2 Department of Geriatric,Tongji Hospital, Tongji Medical College of Huazhong University of Science and Technology, Hubei, 430030 Wuhan People’s Republic of China

**Correction: Cancer Cell Int (2022) 22:7**

10.1186/s12935-021-02427-4

In this article [1], Fig. 2f appeared incorrectly and have now been corrected in the original publication. For completeness and transparency, the incorrect and correct versions are displayed below. The original article has been corrected.

**Incorrect Figure 2**:

**Fig. 2:**
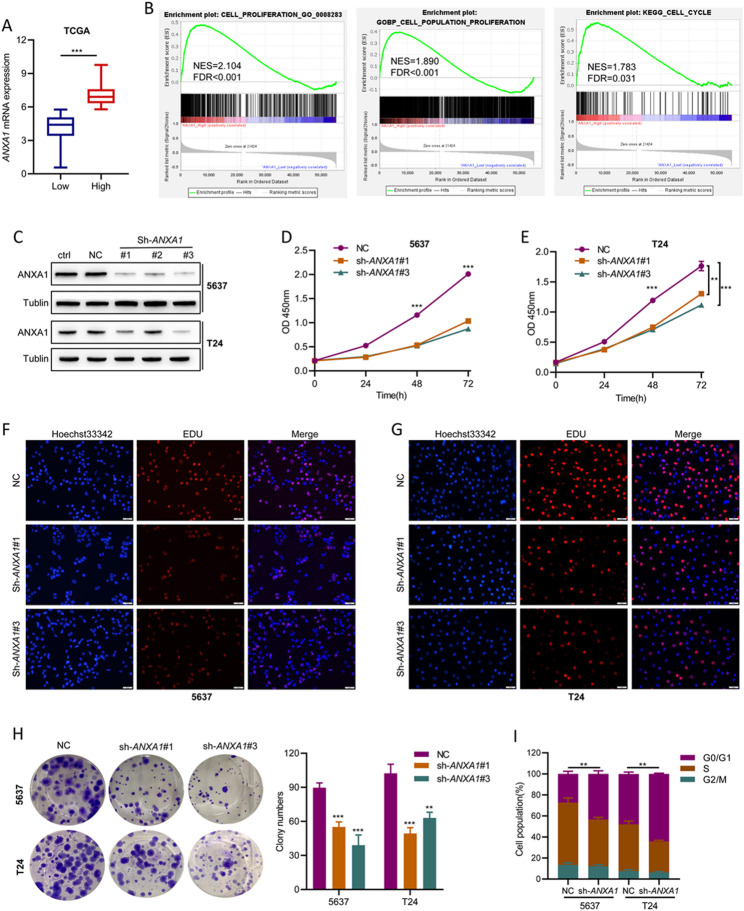
*ANXA1* knockdown impairs proliferation, colony formation, and the G1-to-S phase transition in BLCA cell lines. A *ANXA1* expression levels in high *ANXA1* samples compared with their low *ANXA1* counterparts among 414 BLCA samples in the TCGA cohort. The optimal cutoff value of low and high ANXA1 was calculated by X-tile software. B GSEA analysis of proliferation and cell cycle target gene sets based on the TCGA cohort. C Western blot of ANXA1 in BLCA cell lines transfected with negative control sh-RNA and three different sh-RNAs specific to *ANXA1*. Ctrl: normal cells; NC: cells transfected negative control sh-RNA; sh-*ANXA1*#1–3: cells transfected three different sh-RNAs specific to *ANXA1*. D, E A CCK-8 assay was conducted to detect the effect of ANXA1 on cell viability. F, G DNA synthesis in BLCA cells of the NC and sh-*ANXA1* groups was examined by EdU assay. H Colony formation assay of NC and sh-*ANXA1* groups. I Cell cycle assay of NC and sh-*ANXA1* groups. *p < 0.05, ** *p* < 0.01, *** *p* < 0.001

**Correct Figure 2**:

**Fig. 2:**
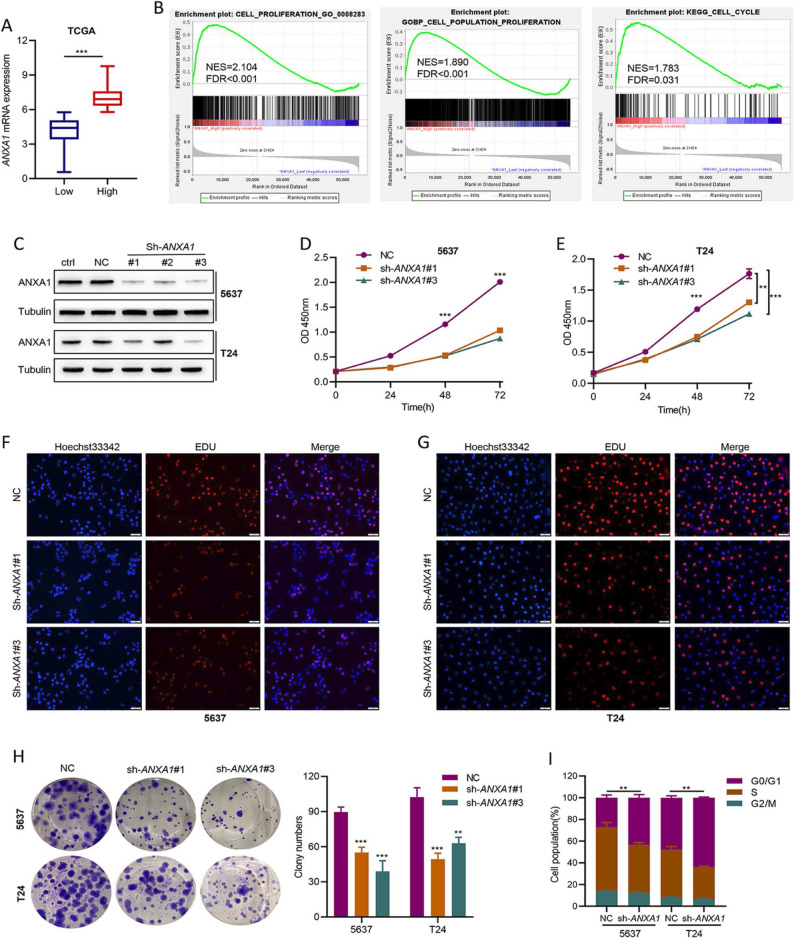
*ANXA1* knockdown impairs proliferation, colony formation, and the G1-to-S phase transition in BLCA cell lines. A *ANXA1* expression levels in high *ANXA1* samples compared with their low *ANXA1* counterparts among 414 BLCA samples in the TCGA cohort. The optimal cutoff value of low and high ANXA1 was calculated by X-tile software. B GSEA analysis of proliferation and cell cycle target gene sets based on the TCGA cohort. C Western blot of ANXA1 in BLCA cell lines transfected with negative control sh-RNA and three different sh-RNAs specific to *ANXA1*. Ctrl: normal cells; NC: cells transfected negative control sh-RNA; sh-*ANXA1*#1–3: cells transfected three different sh-RNAs specific to *ANXA1*. D, E A CCK-8 assay was conducted to detect the effect of ANXA1 on cell viability. F, G DNA synthesis in BLCA cells of the NC and sh-*ANXA1* groups was examined by EdU assay. H Colony formation assay of NC and sh-*ANXA1* groups. I Cell cycle assay of NC and sh-*ANXA1* groups. *p < 0.05, ** *p* < 0.01, *** *p* < 0.001.
