# Supplementary figures and images for: Annexin A1 promotes the progression of bladder cancer via regulating EGFR signaling pathway
Source: Cancer Cell Int. 2022 Jan 6;22:7. doi: 10.1186/s12935-021-02427-4 (PMC8740017; doi:10.1186/s12935-021-02427-4)

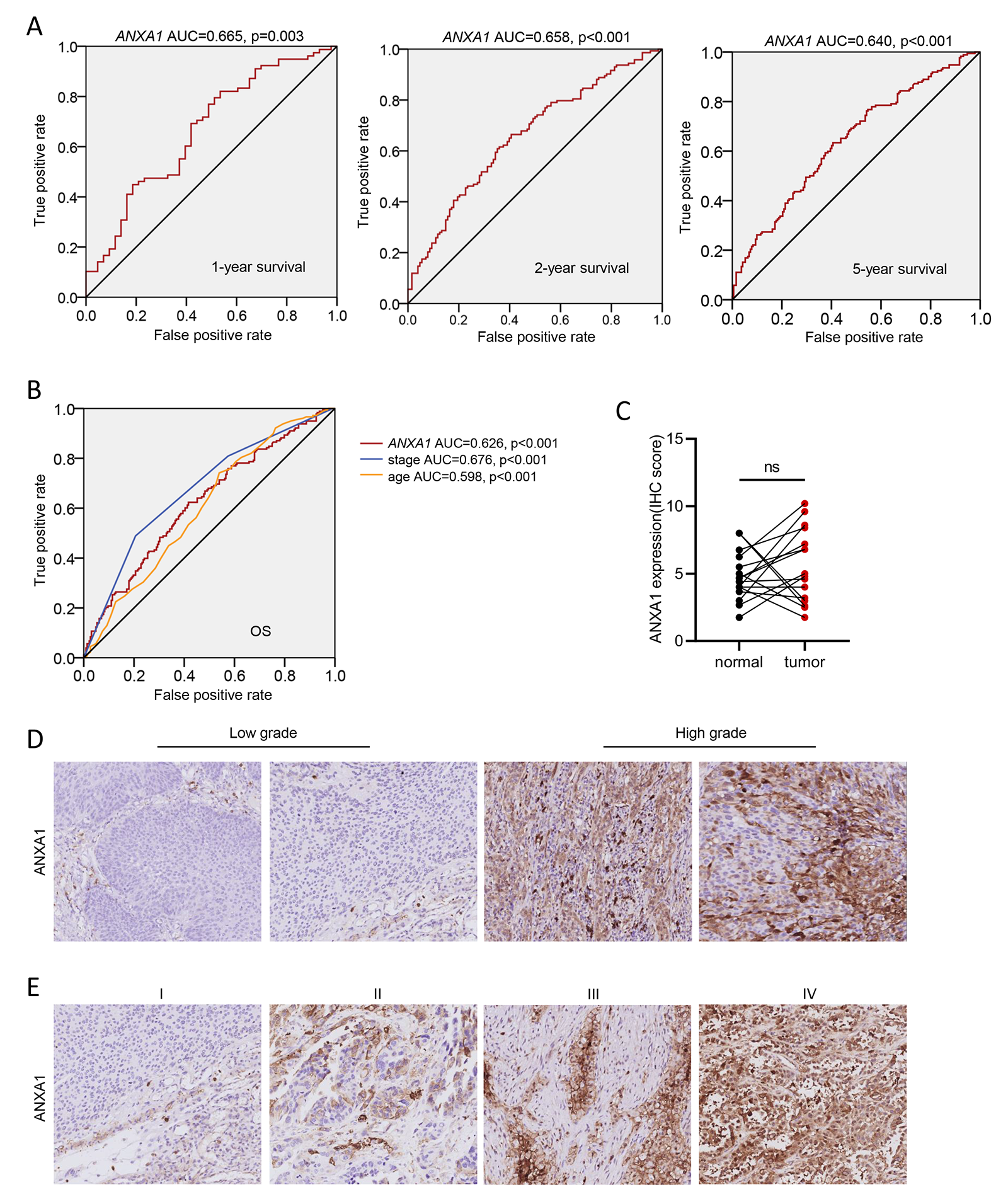

Supplement: Supplementary file 1 — Additional file 1. Figure S1. (A) The ROC curves for the signature in predicting survival at time points of 1-, 2-, and 5-year in the TCGA dataset. (B) The ROC curves comparing the prognostic values of risk score and several clinical factors in the TCGA dataset. (C) The expression level of ANXA1 in tumor tissues and adjacent normal tissues of 17 patients with bladder cancer. (D) Representative ANXA1 expression in patients with different histological grades. (Original magnification ×200). (E) Representative ANXA1 expression in patients with different clinical stages. (Original magnification ×200). [file 12935_2021_2427_MOESM1_ESM.tif]

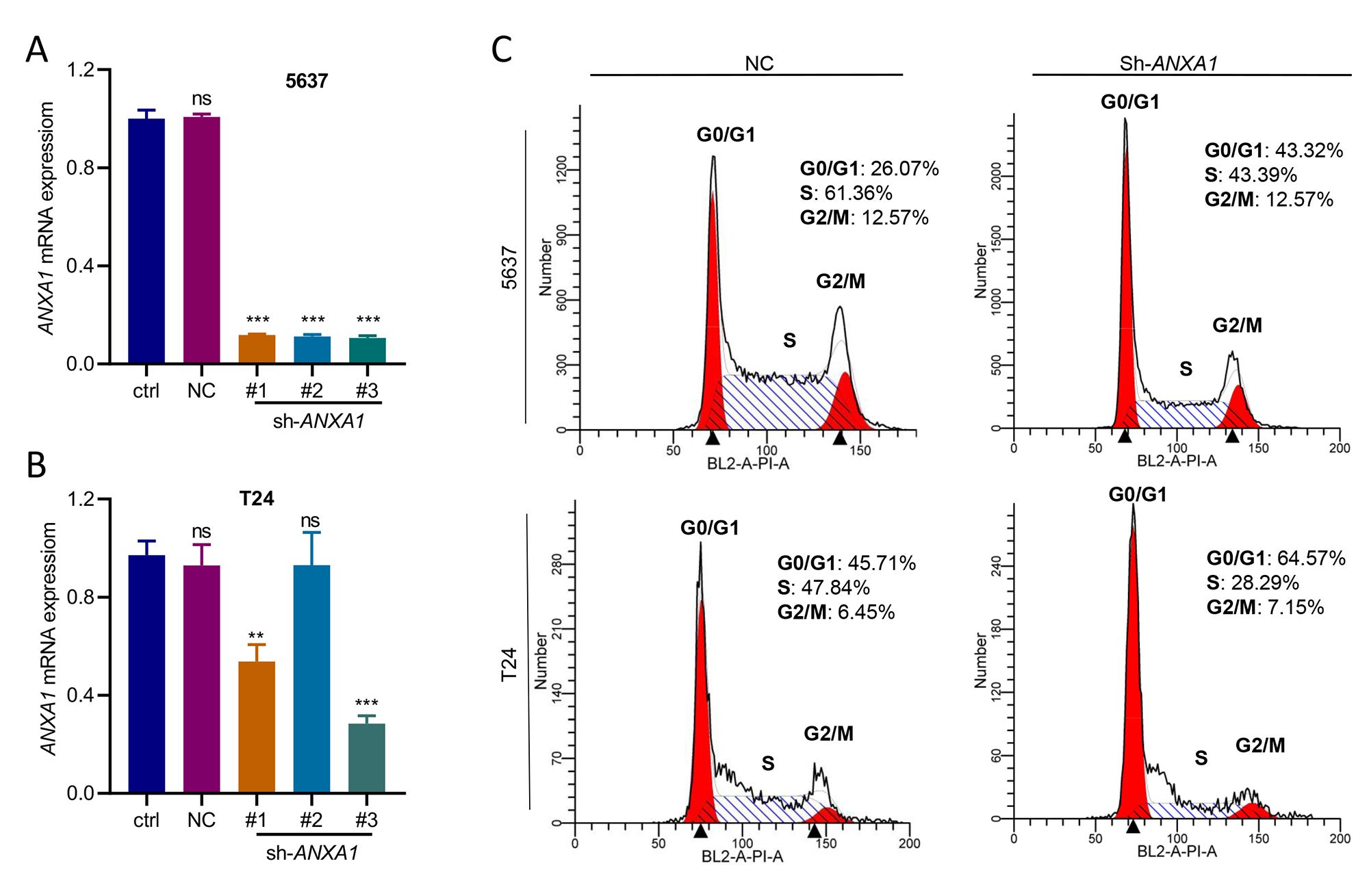

Supplement: Supplementary file 2 — Additional file 2. Figure S2. (A-B) qRT-PCR analysis of ANXA1 in BLCA cell lines transfected negative control shRNA and three different sh-RNAs specific to ANXA1. (C) Cell cycle assay in the NC and sh-ANXA1 groups. [file 12935_2021_2427_MOESM2_ESM.tif]

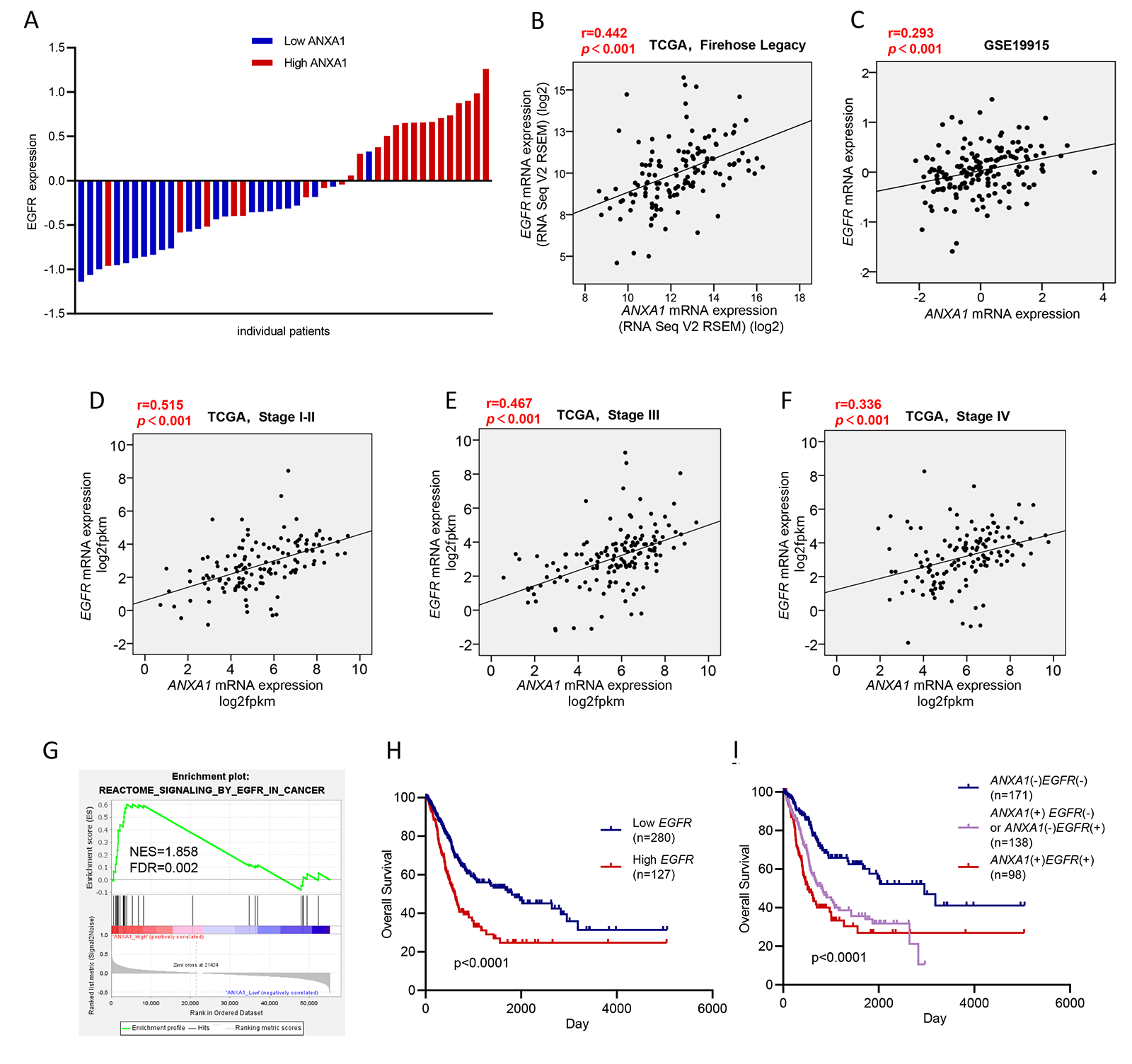

Supplement: Supplementary file 3 — Additional file 3. Figure S3. (A) The waterfall plot displayed the EGFR expression of each sample in the low and high ANXA1 groups. (B-F) Pearson correlation analysis of ANXA1 and EGFR gene expression in TCGA and GEO datasets. (G) GSEA analysis of EGF/EGFR signaling in high and low ANXA1 samples based on the TCGA dataset. (H) Kaplan–Meier survival curves of BLCA patients with high EGFR and low EGFR expression in the TCGA cohort. (I) Kaplan-Meier curve for the prognosis of patients with high and low ANXA1 or EGFR expression levels in the TCGA cohort. [file 12935_2021_2427_MOESM3_ESM.tif]
