# Supplementary material for: Annexin A1 promotes the progression of bladder cancer via regulating EGFR signaling pathway
Source: Cancer Cell Int. 2022 Jan 6;22:7. doi: 10.1186/s12935-021-02427-4 (PMC8740017; doi:10.1186/s12935-021-02427-4)
Supplement: Supplementary file 4 — Additional file 4. Table S1. Primer sequences for qRT-PCR. [file 12935_2021_2427_MOESM4_ESM.docx]

| Table S1. Primer sequences for qRT-PCR | | |
| --- | --- | --- |
| Gene | Forward primer (5'- 3') | Reverse primer (3'-5') |
| *ANXA1* | GCGGTGAGCCCCTATCCTA | TGATGGTTGCTTCATCCACAC |
| *EGFR* | GGTGACCGTTTGGGAGTT | CCTGAATGACAAGGTAGCG |
| *GAPDH* | TGACTTCAACAGCGACACCCA | CACCCTGTTGCTGTAGCCAAA |
